# Paying attention to the outcome of others' actions has dissociated effects on observer's peripersonal space representation and exploitation

**DOI:** 10.1038/s41598-023-37189-8

**Published:** 2023-06-22

**Authors:** Maria Francesca Gigliotti, Angela Bartolo, Yann Coello

**Affiliations:** CNRS, UMR 9193-SCALab-Sciences Cognitives et Sciences Affectives, University of Lille-SHS, Villeneuve d’Ascq, 59000 Lille, France

**Keywords:** Psychology, Human behaviour

## Abstract

The representation of peripersonal space (PPS representation) and the selection of motor actions within it (PPS exploitation) are influenced by action outcomes and reward prospects. The present study tested whether observing the outcome of others' actions altered the observer's PPS representation and exploitation. Participants (observers) performed a reachability-judgement task (assessing PPS representation) before and after having observed a confederate (actors) performing a stimuli-selection task on a touch-screen table. In the stimuli-selection task, the stimuli selected could either yield a reward or not, but the probability to select a reward-yielding stimulus was biased in space, being either 50%, 25% or 75% in the actor’s proximal or distal space. After the observation phase, participants performed the stimuli-selection task (assessing PPS exploitation), but with no spatial bias in the distribution of reward-yielding stimuli. Results revealed an effect of actors’ actions outcome on observers’ PPS representation, which changed according to the distribution of reward-yielding stimuli in the actors’ proximal and distal spaces. No significant effect of actors’ actions outcome was found on observers’ PPS exploitation. As a whole, the results suggest dissociated effects of observing the outcome of others’ actions on PPS representation and exploitation.

## Introduction

Performing object-directed motor actions requires to precisely represent the space where these motor actions can take place, which refers to the peripersonal space^1–5^. The peripersonal space (PPS hereafter) acts as an interface between the body and the environment by allowing the selection of the objects that receive particular attention in relation to an intentional motor purpose^6,7^. The particular feature of this interface is that objects located in PPS are coded through multisensory and sensorimotor integrative processes, two mechanisms which operate in the space near single body parts (e.g., peri-hand, peri-head, peri-trunk and peri-feet PPS^8–10^) as well as in the space around the full body^11^. As highlighted by single-unit electrophysiological studies in macaque monkeys^1,12^, human behavioural^13,14^, neuroimaging^15,16^ and brain-damaged patients studies^17,18^, these mechanisms enhance the multisensory treatment of nearby stimuli, which prepares the motor system to interact with these stimuli. Based on this sensorimotor processing, PPS representation serves two essential functions: selecting potential actions towards incentive objects and protecting the body from approaching hazards^5,19–21^.

In order to fulfil this dual function, the representation of PPS constantly adjusts to the situation encountered and can be thereby modulated by several factors. First, PPS representation is highly sensitive to transient or permanent alterations of the sensorimotor system. As evidence, several studies have shown that using a tool can induce an extension of PPS representation^22,23^. For instance, Bourgeois and collaborators^24^ demonstrated that the distance at which an object is perceived to be within arm reach extended in space after tool use, provided that the tool was sufficiently long to induce a functional extension of the arm. This extension was interpreted as the consequence of the incorporation of the functional aspects of the tool into the users’ body schema^23,25–27^. Such incorporation would be induced by a change in body metrics in the somatosensory cortex^27–29^ that would result in a longer arm internal representation^30,31^.

Contrasting with the effect of tool use, damage to the sensorimotor cortex was found to impair the sensorimotor abilities on the contralateral side of the body and to induce a reduction of PPS representation, irrespectively of the side of the body considered^17^. The same reduction of the PPS representation was observed after restricting arm movements with an arm-splint for 24 h^32^, which is known to affect cortical excitability of the sensorimotor neurons dedicated to limb control^33,34^, resulting in reduced movement accuracy^35^ and coordination^36^. In the same vein, Leclere et al.^37,38^ reported that changing the usual gravito-inertial force field during the performance of an object-directed motor task produced also a shrinking of PPS representation. As a whole, these findings underlined that PPS representation is also highly dependent on the accuracy of inputs coming from the body and of sensorimotor internal models^39^, with the consequence that the reduction in their effectiveness generally leads to a reduction in the representation of PPS.

In addition to the factors inherent to the individual (i.e., sensorimotor representations, internal models and accuracy of body-related inputs), the valence assigned to external stimuli has also been identified to affect PPS representation. Indeed, a reduction of PPS representation was observed when potentially dangerous objects were presented within reaching distance, provided that the object's threatening part (e.g., the needle of a syringe) was directed towards participants’ body^40^. This result was explained by an anticipation of the potential positive/negative consequences of acting towards the dangerous object, leading to a suitable approach/avoidance behaviour and to a consequent adjustment of PPS representation. In line with this view, Coello et al.^41^ went one step further by showing that the expectation of rewarding outcomes from motor actions could also induce a modification of PPS representation (see also^42^). The authors asked participants to manually select a set of visual stimuli presented on a touch-screen table. Once selected, the stimuli could either yield a reward or not. The distribution of reward-yielding stimuli could be equal in the whole workspace or biased towards either the distal or the proximal portion of the workspace. Results showed that, after several manual selections, participants tended to select predominantly the stimuli located in the area of the workspace associated with a higher proportion of reward-yielding stimuli, although this was entirely outside the scope of consciousness. These results extended to manual motor actions what was previously observed in tasks involving visual selection without action^43,44^, confirming that visual attention is preferentially oriented towards the most rewarding stimuli and conversely deviated from stimuli associated with no reward. Moreover, the results by Coello et al.^41^ highlighted that PPS representation seems to rely not only on the sensorimotor properties of the body, but also on reward prospects related to the motor actions performed in the environment.

Beyond the findings summarised above, recent studies addressed the issue of the effect of watching someone performing an action on the observer's PPS representation. For instance, Costantini et al.^45^ showed that PPS representation extended after observing a confederate using a tool, although only when the observer held a functionally and structurally similar tool. The authors concluded that PPS remapping through action observation is modulated by the observer’s possibility to perform compatible actions. By contrast, Galigani et al.^46^ did not find any effect of tool-use observation on the multisensory processing of stimuli within PPS, advocating the importance of integrating sensorimotor feedback to actually induce a remapping of PPS representation. Given the divergent results of these studies, the effect of observing the outcomes of another’s action on the perceiver’s PPS representation remains an open issue. More specifically, it is not known yet whether others' action outcomes remap the observer's PPS representation. Furthermore, it is still unclear whether observing others’ action outcomes influences also PPS exploitation, namely the action selection process within such space (and thus, the way individuals act in their near-body action space). Building on our previous research^41,42^, the aim of the present study was therefore to examine whether observing others’ action outcomes altered the observer’s PPS representation and exploitation, which are the two components of the organisation of goal-directed motor actions.

**Figure 1:**
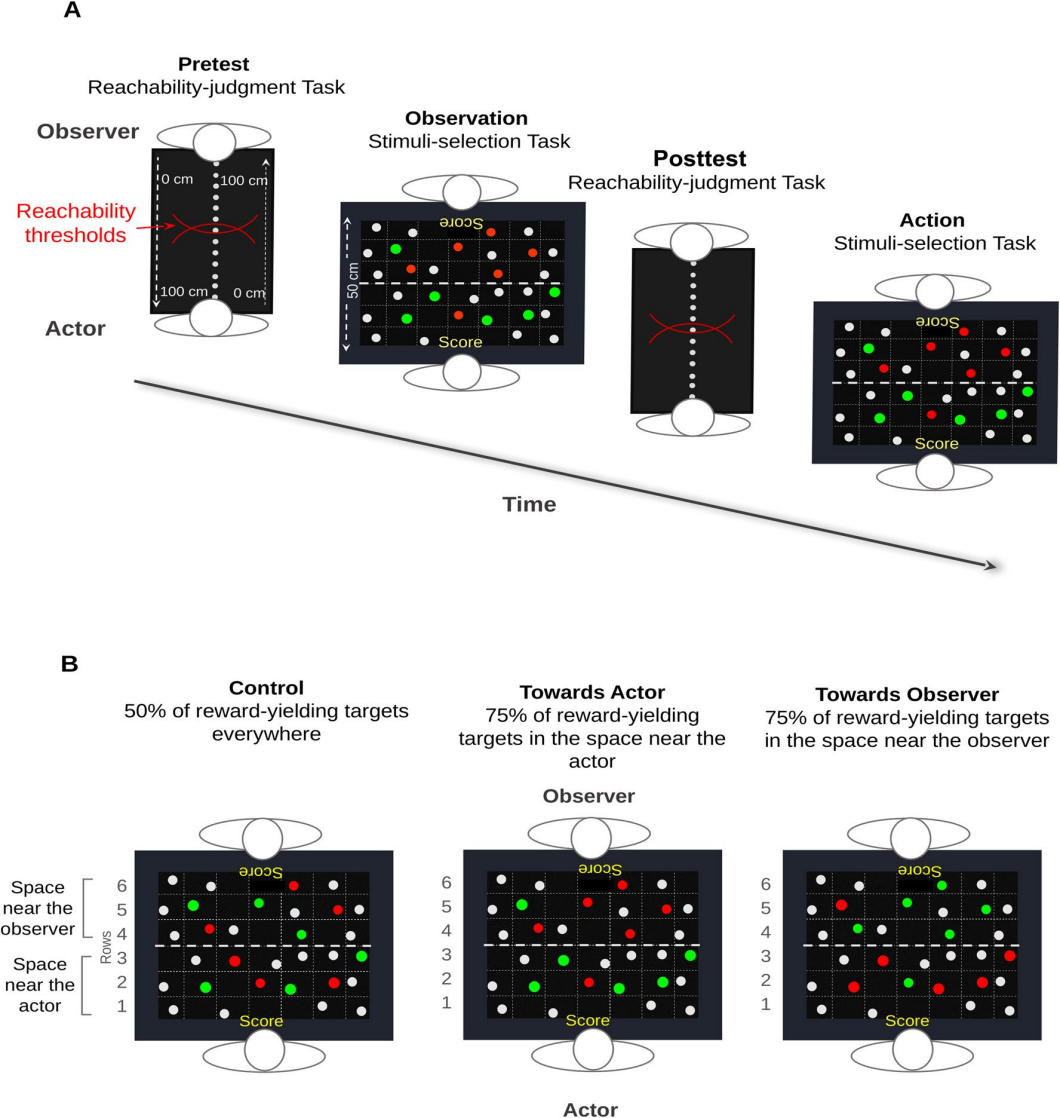
Experimental procedure and tasks. (**A**) Sequential order of the tasks. First, participants performed the reachability-judgment task. Second, the actor performed the stimuli-selection task, while the observer observed his/her confederate’s performance. Third, participants realised the reachability-judgment task for a second time. Finally, participants performed again the stimuli-selection task, but switching their roles: the observer realised the stimuli-selection task, while the actor observed. (**B**) Distribution of the reward-yielding stimuli as function of the group. In the Control group, the probability to select a reward-yielding stimulus was 50% both in the space near the actor (rows 1, 2, 3 of the grid) and in the space near the observer (rows 4, 5, 6). In the Towards Actor group, the probability to select a reward-yielding stimulus was 75% in the space near the actor and 25% in the space near the observer. On the contrary, in the Towards Observer group, it was 75% in the space near the observer and 25% in the space near the actor. It is important to note that when the observers performed the stimuli-selection task, the probability to select a reward-yielding stimulus was 50% both in the near and far spaces (as in the Control Group) for all the three groups, regardless of the group assigned during the observation phase. In this way, we expected observers to base their selection strategy on the observation of actors’ performances, rather than on the detection of a biased distribution of reward-yielding stimuli.

For this purpose, we recruited same-sex dyads of participants and asked them to perform four tasks (Fig. 1A). First, we assessed participants’ PPS representation using a reachability-judgment task (pretest). In this task, participants were requested to estimate whether a set of visual stimuli (1 cm diameter grey dots) randomly presented across 51 distances (0–100 cm from the trunk) appeared reachable when imaging stretching the right arm. Second, participants were randomly assigned the role of actor or observer and performed a stimuli-selection task. This second task was presented as a game that had to be played together, with the aim to reach the highest possible score as a dyad in order to beat the other dyads. The task was first executed by the actors, then by the observers. It consisted in selecting with the right index finger 12 out of 32 stimuli (2.7 cm diameter grey dots) randomly presented on a touch-screen table. Once selected, the stimuli changed their colour from grey to either red (not reward-yielding stimuli) or green (reward-yielding stimuli). The aim of the task for the actor was to find, across the blocks of trials, as many green, reward-yielding stimuli, as possible to get the highest score. Furthermore, the probability of finding a reward-yielding stimulus was manipulated so that it depended on the stimulus location on the touch-screen table (Fig. 1B). In the Control group, the probability to select a reward-yielding stimulus was 50% both in the space near the actor (i.e., rows 1, 2 and 3 on the touch-screen table) and in the space near the observer (i.e., rows 4, 5 and 6). In the Towards Actor group, it was 75% in the space near the actor and 25% in the space near the observer. On the contrary, in the Towards Observer group, it was 75% in the space near the observer and 25% in the space near the actor. Participants performed 17 blocks of 12 stimuli selections (resulting in a total of 204 trials). In the meanwhile, the observers were requested to observe attentively the performance of the actor (observation phase), and were informed that afterwards, they would have to perform the task themselves, contributing thus to the final performance of the dyad. Third, we reassessed the PPS representation (posttest) to test whether the stimuli-selection task performed by the actors had an effect on PPS representation in both the actors and observers. Finally, we asked the observers to perform the stimuli-selection task through 17 supplementary blocks of trials (action phase), to test whether the observation of actors’ performance had an impact on observers’ PPS exploitation. In this last task, the probability to select a reward-yielding stimulus was 50% both in the spaces near the actor and the observer for all participants, regardless of the group assigned during the observation phase. In this way, we expected observers to base their selection strategy on the observation of actors’ performances, rather than on the detection of a biased distribution of reward-yielding stimuli.

We formulated two hypotheses. First, observing the outcome of actors’ actions in the stimuli-selection task should have an effect on the observers’ PPS representation. Precisely, observers should extend or reduce their PPS representation as a function of the area of the workspace associated with more reward-yielding stimuli. Second, the performances of the actors in the stimuli-selection task should have an effect on the observers' PPS exploitation. According to our rationale, when observers were to apply a stimuli selection strategy, they should select more stimuli in the space associated with the largest number of reward-yielding stimuli at the time the stimuli-selection task was performed by the actors.

## Results

The data for 78 same-sex dyads of participants (*N* = 26 in all groups) were collected and analysed. Following outliers’ analysis (see “Methods” section for further details), 75 participants were retained for the both actors and observers’ datasets analysis. First, we analysed the actors’ performance in order to confirm previous findings about the effect of biasing the rewards distribution on the exploitation and representation of PPS^41,42^. Then, we focused on the observers’ performance in order to test our research hypotheses.

### Actor performance

#### Effect of the biased distribution of reward-yielding stimuli on the actor’s PPS representation

As concerns the reachability-judgment task, means, standard deviations and 95% confidence intervals for the reachability thresholds (relative values according to arm-length) obtained in the pretest and posttest sessions for the three groups are presented in Table 1. In the pretest, actors’ mean relative reachability threshold was 0.48 cm (SD = 9.23) on average, which corresponded to an overestimation of 0.67% of participants’ mean actual arm length (M = 71.12 cm, SD = 4.63). First, we tested whether groups’ reachability threshold was equivalent in the pretest. Although this is a common practice, it is however worth noting that the pertinence of testing for baseline differences has been questioned (see^47,48^). In the pretest, the mean reachability threshold was not statistically different in the three groups (Control, Towards Actor, Towards Observer), which were thus homogeneous at the beginning of the task (Control vs Towards Actor: *t*_(47)_ = 0.45, *p* = 0.653, *p*_*adj*_ = 0.764; Control vs Towards Observer: *t*_(50)_ = − 0.30, *p* = 0.764, *p*_*adj*_ = 0.764; Towards Actor vs Towards Observer: *t*_(45)_ = 0.75, *p* = 0.457, *p*_*adj*_ = 0.764).

**Table 1:** Means, standard deviations and 95% confidence intervals for the stimuli-selection task and the reachability-judgment task as function of the group (Control, Towards Observer, Towards Actor) and the role (Actor, Observer).

Group	*N*	Stimuli-selection task			Reachability-judgment task	
		All 17 blocks	First 3 blocks	Last 3 blocks	Pretest	Posttest
		M	M	M	M	M
		SD	SD	SD	SD	SD
		95% CI	95% CI	95% CI	95% CI	95% CI
Actor						
Control	26	5.68	5.42	5.68	0.59 cm	− 0.06 cm
		1.28	1.84	1.32	9.35	9.05
		[5.16, 6.19]	[4.68, 6.16]	[5.15, 6.21]	[− 3.71, 3.60]	[− 3.71, 3.60]
Towards Actor	23	3.68	3.87	3.55	− 0.64 cm	− 0.88 cm
		1.73	2.18	1.90	0.67	10.95
		[2.93, 4.52]	[2.93, 4.81]	[2.73, 4.73]	[− 4.83, 3.54]	[− 5.62, 3.85]
Towards Observer	26	6.42	5.62	7.31	1.36 cm	− 4.6 cm
		1.97	1.93	2.27	8.89	9.26
		[5.62, 7.22]	[4.84, 6.39]	[6.39, 8.23]	[− 2.27, 4.98]	[0.92, 8.40]
Observer						
Control	25	5.43	5.25	5.40	0.18 cm	− 1.62 cm
		1.42	1.95	1.74	7.48	7.72
		[4.84, 6.01]	[4.45, 6.06]	[4.68, 6.12 ]	[− 2.90, 3.27]	[− 4.81, 1.56]
Towards Actor	25	5.17	4.92	4.92	0.313 cm	2.67 cm
		1.27	1.69	1.95	10.55	12.17
		[4.65, 5.69]	[4.22, 5.62]	[4.12, 5.72]	[− 4.04, 4.67]	[− 2.35, 7.70]
Towards Observer	25	5.52	5.79	5.76	2.65 cm	2.83 cm
		1.15	1.69	1.47	12.34	13.06
		[5.05, 6.00]	[5.09, 6.48]	[5.15, 6.37]	[− 2.44, 7.74]	[− 2.56, 8.22]

In the stimuli-selection task, values refer to the mean number of stimuli selected by participants in their distal space. In the reachability-judgment task, values refer to the reachability threshold (cm) reported by participants.

**Figure 2:**
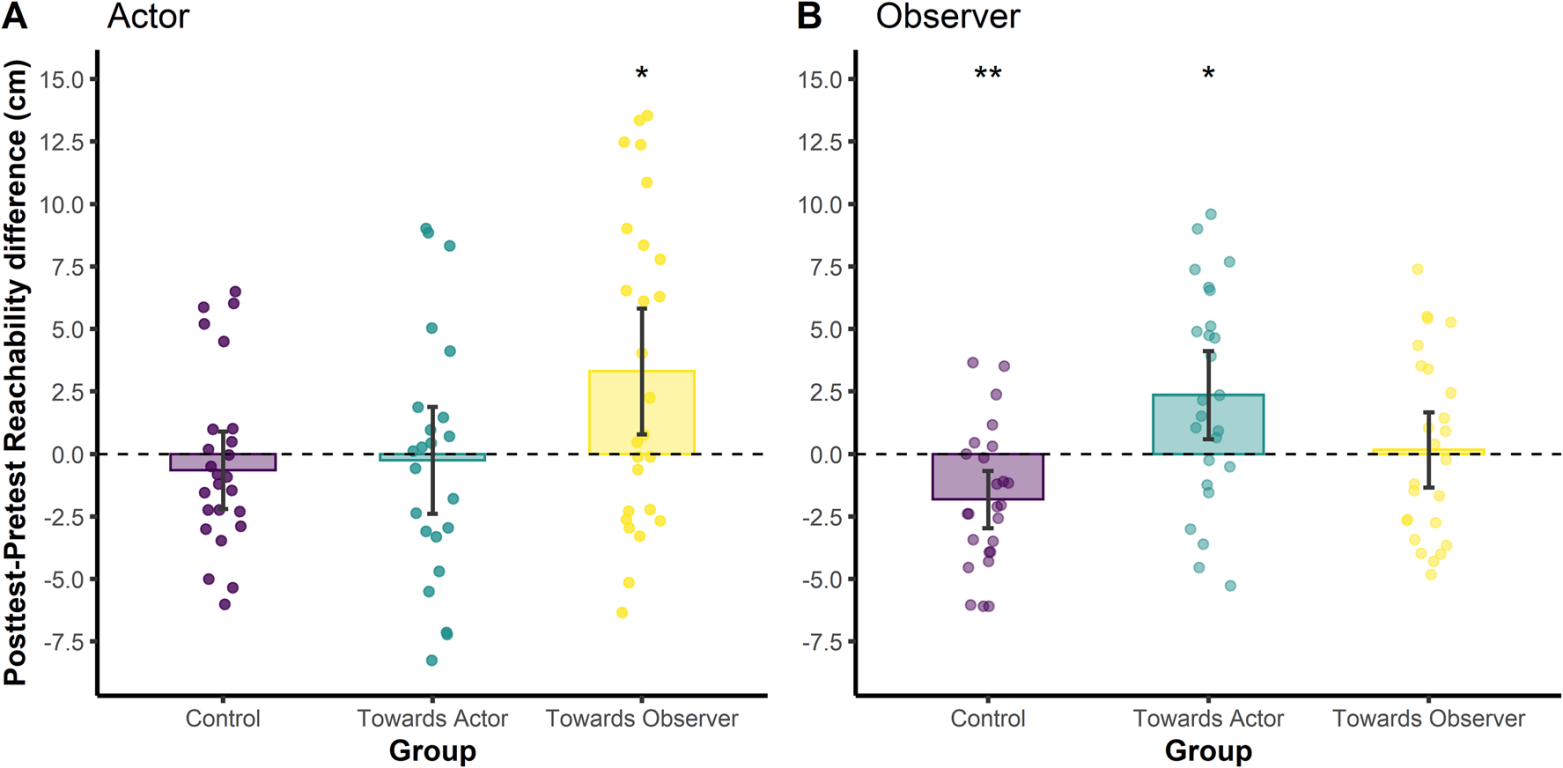
Posttest–pretest difference in reachability threshold as function of the group (Control, Towards Actor, Towards Observer) and the role (Actor, Observer). (**A**) Actor’s posttest–pretest differences in reachability threshold. Only the Towards Observer group showed a significant change in reachability threshold, which increased in the posttest compared to the pretest (posttest–pretest > 0). No significant change was observed for the other two groups. (**B**) Observer’s posttest–pretest differences in reachability threshold. The Towards Actor Group showed a significant change in reachability threshold, which increased in the posttest compared to the pretest (posttest–pretest difference > 0). The Control group showed also a significant change in reachability threshold, which decreased in the posttest compared to the pretest (posttest–pretest difference < 0). No significant change was observed for the Towards Observer group. Histograms represent the mean posttest–pretest difference in reachability threshold. Dots represent individual posttest–pretest differences. Error bars represent 95% confidence intervals. *p < 0.050, **p < 0.010.

Reachability thresholds were statistically compared using a 2-way Session × Group mixed ANOVA, with the Session (Pretest, Posttest) as within-subjects factor and the Group (Control, Towards Actor, Towards Observer) as between-subjects factor. Statistical analysis showed no significant effect of Group (*F*_(2, 72)_ = 1.12, *p* = 0.330, *η*^2^*p*  = 0.03) or Session (*F*_(1, 72)_ = 1.87, *p* = 0.175, *η*^2^*p*  = 0.25). On the contrary, it revealed a significant Session × Group interaction (*F*_(2, 72)_ = 4.65, *p* = 0.013, *η*^2^*p*  = 0.11 indicating a medium effect). More specifically, pairwise comparisons showed that the reachability threshold increased significantly in the posttest compared to the pretest in the Towards Observer group (M = 3.31 cm, SD = 6.23, 95% CI [0.79, 5.82]; *t*_(25)_ = 2.71, *p* = 0.012, *p*_*adj*_ = 0.031), but no significant change was observed neither in the Towards Actor group (M = − 0.24 cm, SD = 4.95, 95% CI [− 2.38, 1.90], *t*_(22)_ = − 0.23*, p* = 0.818, *p*_*adj*_ = 0.904) nor in the Control group (M = − 0.64 cm, SD = 3.84, 95% CI [− 2.19, 0.91], t_(25)_ = − 0.85, p = 0.402, *p*_*adj*_ = 0.563, see Fig. 2A).

#### Effect of the biased distribution of reward-yielding stimuli on the actor’s PPS exploitation

**Figure 3:**
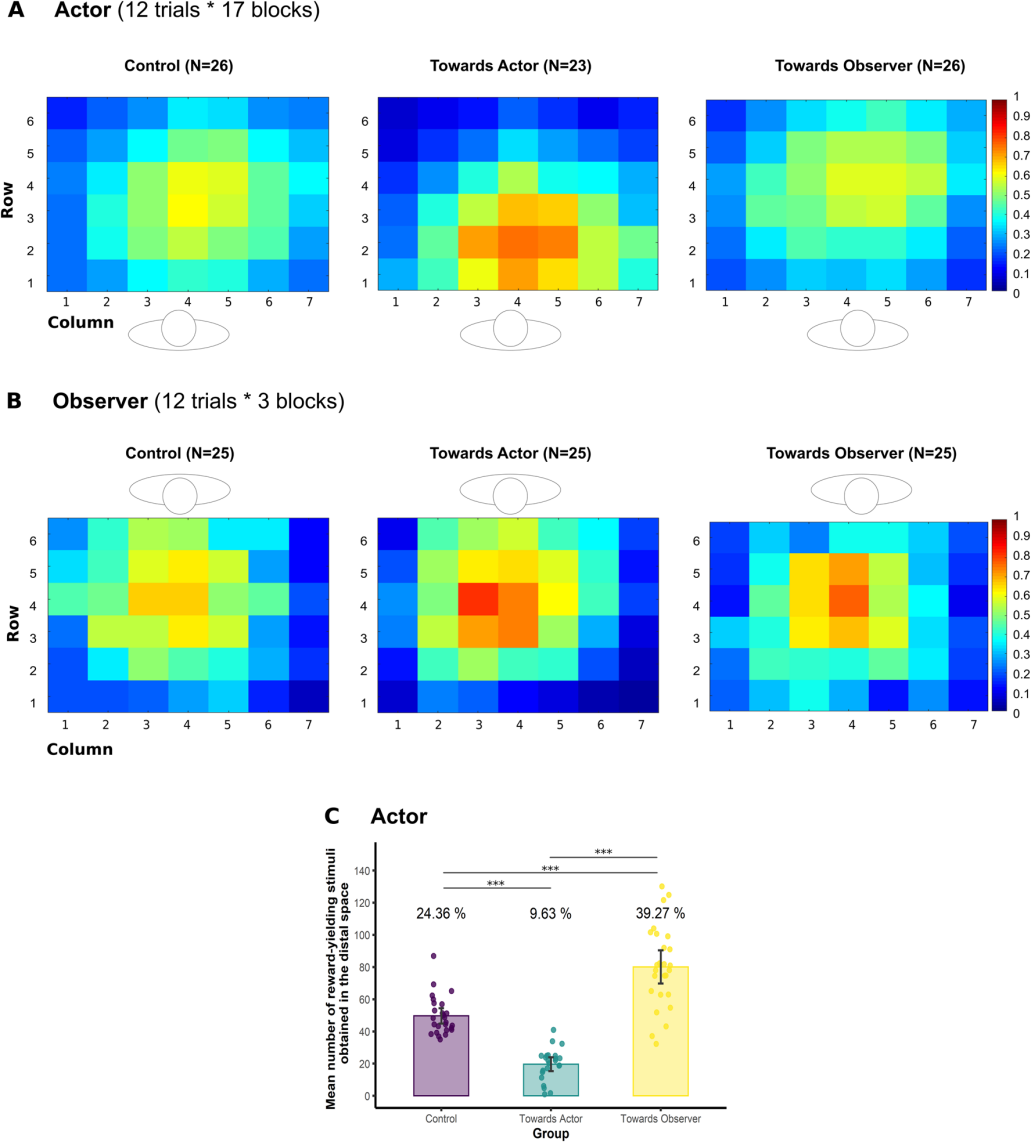
Density maps illustrating the frequency at which each location of the touch-screen table was chosen when it contained a stimulus. The rectangles represent the distribution grid composed of 42 cells (6 rows × 7 columns). The colour bar ranges from blue (rare selection) to red (frequent selection). The human silhouette above or below each density map represents participants’ position during the task. (**A**) Actors’ performance during the stimuli-selection task. The Control group tended to explore the whole surface. The Towards Actor group explored mainly the space near themselves, while the Towards Observer group explored the whole surface tending slightly towards the space near the observer. (**B**) Observers’ performance during the stimuli-selection task (first 3 blocks only). The three groups did not show any particular trend in their early selection strategy. (**C**) Mean number of reward-yielding stimuli obtained by the actor. Histograms represent the mean number of reward-yielding stimuli obtained by the actor in the distal space (i.e., near the observer). Dots represent individual data and error bars 95% confidence intervals. Percentage values represent the proportion of reward-yielding stimuli obtained in the distal space with respect to the total amount of stimuli selected. ***p < 0.001.

As regards the stimuli-selection task, actors selected overall more stimuli in the area of the touch-screen table that was associated with a higher number of reward-yielding stimuli. Figure 3A illustrates the frequency at which each location of the touch-screen table was selected when it contained a stimulus during all the 17 blocks. Participants obtained thereby more rewards in the area exploited, except the Control group for which reward-yielding stimuli were equally distributed in space. To analyse actors’ performances in the stimuli-selection task, we pooled rows 1-2-3 to delimit the space near the actors, and rows 4-5-6 to delimit the space near the observers. As the two spaces were complementary, statistical analyses were performed only on the stimuli selected in the space near the observers, which constituted also, for the actors, an indicator of the tendency to invade other’s PPS. Mean, standard deviation and 95% confidence interval values for each variable measured in the stimuli-selection task are presented in Table 1.

First, we assessed whether actors explored the space associated with a higher number of reward-yielding stimuli depending on the group. For this purpose, we computed, per each participant in each group, the mean number of stimuli selected across all blocks in the space near the observers (i.e., actors’ distal space). The three groups were compared using a Kruskal–Wallis test with the Group (Control, Towards Actor, Towards Observer) as between-subjects factor. Statistical analysis revealed a significant effect of the Group (*H*_(2, 75)_ = 24.68, *p* < 0.001,* ϵ*^2^_*H*_ = 0.31, indicating a large effect). Dunn’s test for pairwise comparisons showed that the Towards Observer group selected more stimuli (M = 6.42, SD = 1.97) in the space near the observer than the Towards Actor group (M = 3.68, SD = 1.73; *Z* = − 4.90, *p* < 0.001, *p*_*adj*_ < 0.001), but not than the Control group (M = 5.68, SD = 1.28; *Z* = 1.68, *p* = 0.092, *p*_*adj*_ = 0.176). On its turn, the Control group selected more stimuli in the space near the observer than the Towards Actor group (*Z* = − 3.27, *p* = 0.001, *p*_*adj*_ = 0.007, see Table 1). Figure 3C shows the mean number of reward-yielding stimuli obtained by the actor in their distal space.

Second, we analysed how actors’ selection strategy changed at the end when compared to the beginning of the task depending on the group. For that purpose, we computed, per each participant, the difference between the mean number of stimuli selected in the space near the observer in the last 3 and in the first 3 blocks of stimuli selections. The three groups were compared by the means of a one-way Welch’s ANOVA, with the Group (Control, Towards Actor, Towards Observer) as between-subjects factor. Results revealed a significant effect of Group (*F*_(2, 75)_ = 16.5, *p* < 0.001, ω^2^ = 0.33 indicating a large effect). Wilcoxon signed-rank test for paired samples showed that the Towards Observer group selected statistically more stimuli in the space near the observer at the end compared to the beginning of the task (*Z* = 65, *p* = 0.009, *p*_*adj*_ = 0.031, *r* = 0.50 indicating a large effect). By contrast, no significant change was observed for the Towards Actor group (*Z* = 143, *p* = 0.603, *p*_*adj*_ = 0.791, *r* = 0.11), nor for the Control group (*Z* = 145, *p* = 0.647, *p*_*adj*_ = 0.799, *r* = 0.09, see Table 1).

**Figure 4:**
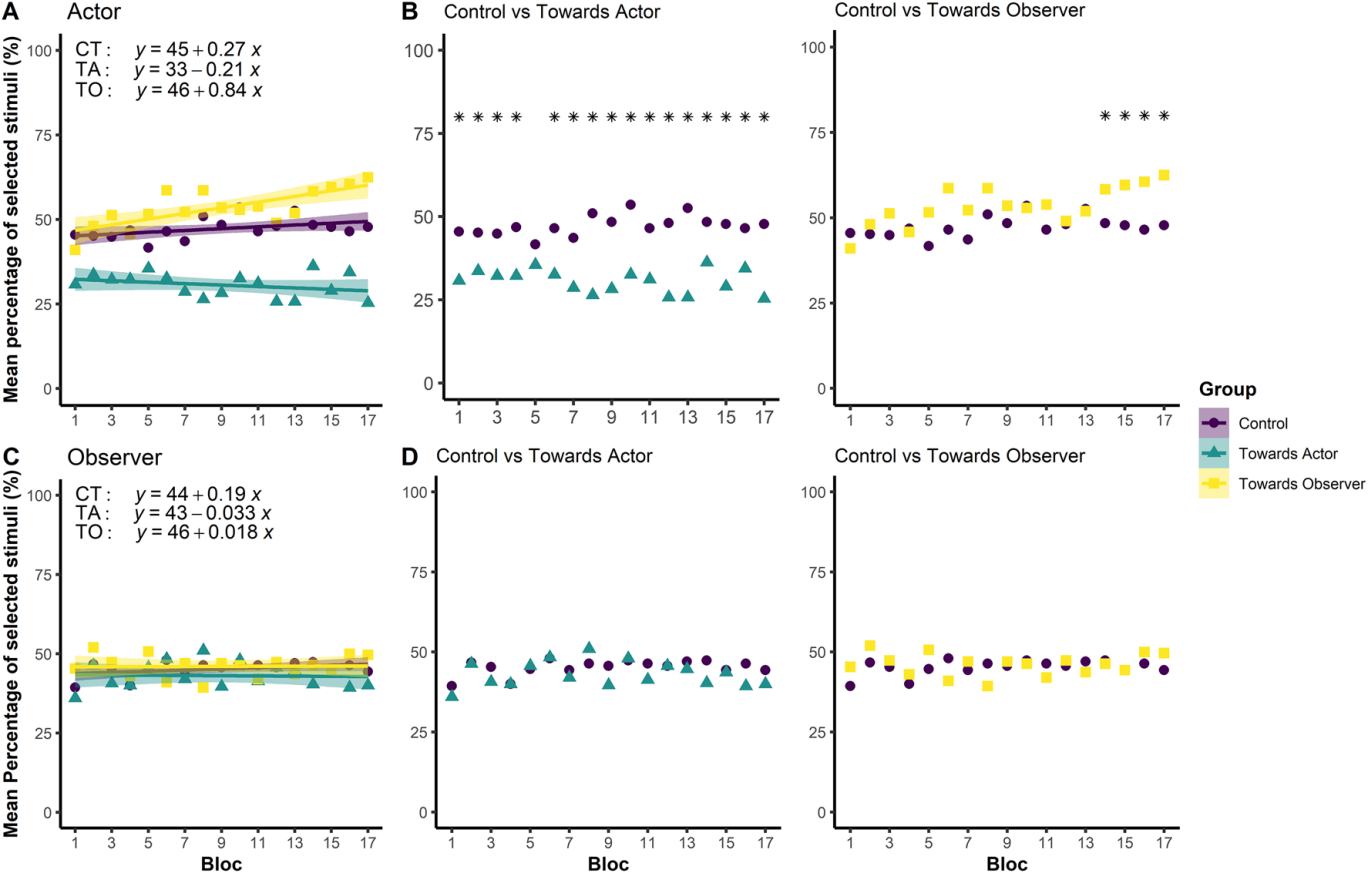
Mean percentage of stimuli selected in the space near the observer (for the actor) or near the actor (for the observer), as function of the group (Control, Towards Actor, Towards Observer) and the role (Actor, Observer). (**A**) Mean percentage of stimuli selected by the actor in the space near the observer across the 17 blocks, as function of the group. As shown by linear regressions, only the Towards Observer group changed its selection strategy during the task: they selected progressively more stimuli in the space near the observer, which was associated, for this group, to a higher probability of obtaining a reward-yielding stimulus. (**B**) Results of the permutation-based multiple comparisons tests for the actor (*p < 0.050). The Towards Actor and Control groups showed different selection strategies from the 1st block on, and throughout all the task. By contrast, the Towards Observer group showed a different strategy from the Control group from the 14th block on. (**C**) Mean percentage of stimuli selected by the observer in the space near the actor across the 17 blocks, as function of the group. As shown by linear regressions, the selection strategy adopted by the three groups did not change across the task. (**D**) Results of the permutation-based multiple comparisons for the observer. Any difference did not emerge between the three groups. *p < 0.050.

Third, we wanted to identify the precise moment at which the change in the exploration strategy occurred. For that purpose, we computed, per each participant in each group, the mean number of stimuli selected in the space near the observer in each of the 17 blocks. When considering all the 17 blocks, regression analysis revealed a progressive increase of the number of stimuli selected in the space near the observer in the Towards Observer group (*R* = 0.74, *F*_(1,15)_ = 18.21, *p* < 0.001, *p*_*adj*_ = 0.007) across the blocks. On the contrary, no significant change in the selection strategy was found in the Towards Actor (*R* = 0.30, *F*_(1,15)_ = 1.53, *p* = 0.234, *p*_*adj*_ = 0.378) and Control (*R* = 0.45, *F*_(1,15)_ = 3.78, *p* = 0.071, *p*_*adj*_ = 0.166; see Fig. 4A) groups. When looking at the precise moment at which the change in the exploration strategy became significant, permutation-based multiple comparisons revealed that the Towards Observer group’s strategy started to diverge from the one of the Control group from the 14th block on (see Fig. 4B). By contrast, no change in selection strategy emerged for the Towards Actor group when contrasted to the Control group, the two groups following a stable and consistent strategy all along the task (see Fig. 4B). All mean, *Z* and *p* values in relation to permutation-based multiple comparisons are reported in Table 2.

**Table 2:** Mean percentage of stimuli selected by each group in the space near the observer (for the actor) and in the space near the actor (for the observer), and z and p values returned by permutation-based multiple comparisons.

Block	*M*_CT_	(vs)	*M*_TA_	*z*	*p*	*M*_CT_	(vs)	*M*_TO_	*z*	*p*
Actor										
1	45.51		30.80	2.24	0.026*	45.51		41.03	0.79	0.463
2	45.19		33.70	2.08	0.039*	45.19		48.08	− 0.56	0.620
3	44.87		32.25	2.45	0.013*	44.87		51.28	− 1.20	0.255
4	46.79		32.25	2.28	0.021*	46.79		45.83	0.16	0.917
5	41.67		35.51	1.00	0.321	41.67		51.60	− 1.61	0.119
6	46.47		32.61	2.31	0.018*	46.47		58.65	− 2.02	0.049*
7	43.59		28.62	2.42	0.017*	43.59		52.24	− 1.59	0.131
8	50.96		26.45	3.49	0.000***	50.96		58.65	− 1.22	0.243
9	48.40		28.26	3.58	0.000***	48.40		53.53	− 0.88	0.418
10	53.53		32.61	3.34	0.000***	53.53		52.88	0.10	0.959
11	46.47		31.16	2.77	0.006**	46.47		53.85	− 1.32	0.215
12	48.08		25.72	3.35	0.001**	48.08		49.04	− 0.14	0.923
13	52.56		25.72	4.00	0.000***	52.56		51.92	0.10	0.963
14	48.40		36.23	2.23	0.027*	48.40		58.33	− 2.09	0.039*
15	47.76		7.15	3.13	0.001**	47.76		59.62	− 2.03	0.046*
16	46.47		7.27	2.52	0.011*	46.47		60.58	− 2.62	0.009**
17	47.76		7.50	4.03	0.000***	47.76		62.50	− 2.89	0.004**
Observer										
1	39.33		36.00	0.63	0.578	39.33		45.33	− 1.07	0.309
2	46.67		46.33	0.06	1.000	46.67		52.00	− 0.91	0.402
3	45.33		40.67	1.04	0.339	45.33		47.33	− 0.48	0.698
4	40.00		40.00	0.00	1.000	40.00		43.00	− 0.60	0.600
5	44.67		45.67	− 0.21	0.889	44.67		50.67	− 1.29	0.227
6	48.00		48.33	− 0.09	1.000	48.00		41.00	1.62	0.126
7	44.33		42.00	0.40	0.740	44.33		47.00	− 0.40	0.732
8	46.33		51.00	− 0.87	0.429	46.33		39.33	1.15	0.271
9	45.67		39.67	1.32	0.221	45.67		47.00	− 0.36	0.787
10	47.33		48.00	− 0.15	0.942	47.33		46.33	0.26	0.859
11	46.33		41.33	1.02	0.350	46.33		42.00	0.88	0.417
12	45.67		45.67	0.00	1.000	45.67		47.33	− 0.28	0.835
13	47.00		44.67	0.40	0.735	47.00		43.67	0.57	0.610
14	47.33		40.33	1.07	0.309	47.33		46.33	0.16	0.917
15	44.33		43.67	0.13	0.947	44.33		44.33	0.00	1.000
16	46.33		39.33	1.03	0.334	46.33		50.00	− 0.57	0.609
17	44.33		40.00	0.77	0.478	44.33		49.67	− 1.10	0.309

*MCT* mean percentage of stimuli selected by the Control group, *MTA* mean percentage of stimuli selected by the Towards Actor group, *MTO* mean percentage of stimuli selected by the Towards Observer group.*p < 0.050, **p < 0.010, ***p < 0.001.

Taken as a whole, the results showed that the Towards Actor group selected more stimuli in their near space all along the task, and this since the first 3 blocks. Thus, we could say that the Towards Actor group learned rapidly and was influenced by the biased distribution of reward-yielding stimuli early in the task. On the contrary, the Towards Observer group needed more time to be influenced by the biased distribution of reward-yielding stimuli. As shown in Table 1 and Fig. 4B, the number of reward-yielding stimuli was always higher, and statistically significantly so, in the Towards Observer group than in the Control only in the last 4 blocks.

### Observer performance

#### Effect of the biased distribution of reward-yielding stimuli on the observer’s PPS representation

As regards the reachability-judgment task, mean, standard deviation and 95% confidence interval values for the observers’ reachability thresholds obtained in the pretest and posttest sessions for the three groups appear in Table 1. In the pretest, observers’ mean relative reachability threshold was 1.05 cm (SD = 10.20) on average, which corresponded to an overestimation of 1.46% of participants’ mean actual arm length (M = 71.73 cm, SD = 4.63). The mean reachability threshold obtained in the pretest was not statistically different in the three groups (Control, Towards Actor, Towards Observer), which were thus homogeneous at the beginning of the task (Control vs Towards Actor: *t*_(48)_ =  −0.05, *p* = 0.961, *p*_*adj*_ = 0.961; Control vs Towards Observer: *t*_(48)_ = − 0.85, *p* = 0.397, *p*_*adj*_ = 0.713; Towards Observer vs Towards Actor: *t*_(48)_ = 0.72, *p* = 0.475, *p*_*adj*_ = 0.713).

Reachability thresholds were statistically compared using a 2-way Session × Group mixed ANOVA, with the Session (Pretest, Posttest) as within-subjects factor and the Group (Control, Towards Actor, Towards Observer) as between-subjects factor. Statistical analysis showed no significant effect of Group (*F*_(2, 72)_ = 0.68, *p* = 0.511, *η*^2^*p*  = 0.02) or Session (*F*_(1, 72)_ = 3.34, *p* = 0.562, *η*^2^*p*  = 0.01). On the contrary, it revealed a significant Session × Group interaction (*F*_(2, 72)_ = 8.33, *p* < 0.001, *η*^2^*p* = 0.19 indicating a large effect). More precisely, pairwise comparisons showed that the reachability threshold decreased significantly in the posttest compared to the pretest in the Control group (M = − 1.81 cm, SD = 2.78, 95% CI [− 2.96, − 0.66]; *t*_(24)_ =  − 3.25, *p* = 0.003, *p*_*adj*_ = 0.016), and increased significantly in the Towards Actor group (M = 2.36 cm, SD = 4.25, 95% CI [0.60, 4.11]; *t*_(24)_ = 2.77*, p* = 0.011, *p*_*adj*_ = 0.031). No significant difference between the pretest and the posttest was observed in the Towards Observer group (M = 0.18 cm, SD = 3.65, 95% CI [− 1.33, 1.69]; *t*_(24)_ = 0.24, *p* = 0.810, *p*_*adj*_ = 0.904 see Fig. 2B).

Taken as a whole, these results show a symmetric effect of reward-yielding stimuli on PPS representation for the actor and the observer: for both of them, reward-yielding stimuli induced an increase of PPS representation only when they were located in the distal space (i.e., in the space near the observer for the actor, and in the space near the actor for the observer).

Finally, we carried out a complementary analysis in order to test for a potential decay in the posttest of the effect of observing others’ outcomes on the observers’ PPS representation before the execution of the stimuli-selection task. For this purpose, we compared the reachability thresholds computed after splitting the first vs. second half of the posttest trials, all groups considered together. Wilcoxon signed-rank test showed a non-significant change in reachability thresholds between the first and the second part of the reachability task (First half: M = 0.67 cm, SD = 11.55; Second half: M = 1.69 cm, SD = 11.63;* Z* = 1701, *p* = 0.062). No evidence for a potential decay emerged either when analysing the three groups separately. Results showed indeed no significant difference for the Control (First half: M = − 1.01 cm, SD = 9.17; Second half: M = − 2.29 cm, SD = 6.92; *Z* = 206, *p* = 0.252, *p*_*adj*_ = 0.378) and the Towards Observer (First half: M = 1.35 cm, SD = 13.11; Second half: M = 3.54 cm, SD = 13.85; *Z* = 99, *p* = 0.090, *p*_*adj*_ = 0.176) groups and a significant increase in the second half compared to the first one in the Towards Actor group (First half: M = 1.53 cm, SD = 12.36; Second half: M = 3.91 cm, SD = 12.44;* Z* = 65, *p* = 0.007, *p*_*adj*_ = 0.029, r = 0.525 indicating a large effect). This last result suggests that the effect of observing others is rather stronger at the end compared to the beginning of the task, and appears thereby opposite to the hypothesis of a decay. It also underlines that the temporal aspects of reachability-judgment adjustment should be subject to greater scrutiny in future studies.

#### Effect of the biased distribution of reward-yielding stimuli on the observer’s PPS exploitation

To analyse the observers’ performance at the stimuli-selection task, as for the actor we pooled rows 1-2-3 to define the space near the actor, and rows 4-5-6 to delimit the space near the observer. Statistical analyses of the observers’ performances were conducted on the stimuli selected in the space near the actor.

First, we assessed whether the observers, depending on the group, exploited preferentially the space that was associated with a higher number of reward-yielding stimuli when the actor performed the task. For that purpose, we computed, per each participant in each group, the mean number of stimuli selected across all blocks in the space near the actor. The three groups were compared through a one-way ANOVA, performed using the Group (Control, Towards Actor, Towards Observer) as between-subjects factor. Statistical analysis revealed no significant effect of the Group (*F*_(2, 72)_ = 0.50, *p* = 0.609, *η*^2^*p* = 0.01), the three groups not differing thus in the mean number of stimuli selected in the space near the actor (5.37 stimuli per block on average, see Table 1).

Second, we tested whether the observers, depending on the group, changed their selection strategy at the end compared to the beginning of the task. Therefore, we computed, per each participant, the difference between the mean number of stimuli selected in the space near the actors in the last 3 blocks and in the first 3 blocks of trials*.* We compared the three groups using a 2-way Session × Group mixed ANOVA, with the Block (First, Last) as within-subjects factor and the Group (Control, Towards Actor, Towards Observer) as between-subjects factor. Statistical analysis did not reveal a significant effect neither of the Group (*F*_(2, 72)_ = 2.03, *p* = 0.138, *η*^2^*p*  = 0.05), nor of the Block (*F*_(1, 72)_ = 0.04, *p* = 0.851, *η*^2^*p*  < 0.01), nor of the Block × Group interaction (*F*_(2, 72)_ = 0.06, *p* = 0.937, *η*^2^*p*  < 0.01). These results were corroborated by regression analysis, which did not show any particular change in the observer’s selection strategy across the task neither in the Control group (*R* = 0.39, *F*_(1,15)_ = 2.75, *p* = 0.118, *p*_*adj*_ = 0.206), nor in the Towards Actor group (*R* = 0.04, *F*_(1,15)_ = 0.03, *p* = 0.871, *p*_*adj*_ = 0.914), nor in the Towards Observer group (*R* = 0.03, *F*_(1,15)_ = 0.01, *p* = 0.921, *p*_*adj*_ = 0.921).

Third, we compared the three groups only in the first 3 blocks, in order to test whether it was possible to find evidence for an early influence of observing actors’ performance on the observers’ selection strategy. Indeed, repeating the task over 17 blocks could have neutralised this effect as, for all the three groups, the probability to select a reward-yielding target was set at 50% in both the near and far space (as in the Control group). Figure 3B illustrates the frequency at which each location of the touch-screen table was selected when it contained a stimulus during the first 3 blocks. We conducted a one-way ANOVA on the mean number of stimuli selected in the space near the actor during the first 3 blocks, using the Group (Control, Towards Actor, Towards Observer) as between-subject factor. Again, results did not reveal any significant effect of the Group (*F*_(2, 72)_ = 1.51, *p* = 0.228, *η*^2^*p*  = 0.04), not allowing to conclude on an early effect of actors’ performance on the observers’ selection strategy.

## Discussion

The aim of the present study was to test whether observing the outcome of others’ actions influenced the observers’ PPS representation and exploitation. The study was inspired by the ones by^41,42^, with dyads of participants performing a reachability-judgment task (to assess PPS representation) and a stimuli-selection task (to assess PPS exploitation). The novelty of the present study was that, in the stimuli-selection task, participants were assigned either the role of the actor or that of the observer. The observer was asked to perform the stimuli-selection task after observing the actor performing it.

As regards the actors’ performances, results replicated previous findings about the effect of the spatial distribution of reward-yielding stimuli on both PPS representation and exploitation. Concerning actor’s PPS exploitation, results showed that, in the stimuli-selection task, participants selected more stimuli in the space associated with a higher number of reward-yielding stimuli. Indeed, results showed that the selection strategy adopted by the Towards Actor (i.e., reward-yielding stimuli in the space near the actor) and the Towards Observer (i.e., reward-yielding stimuli in the space far from the actor and near the observer) differed from the one adopted by the Control Group (i.e., reward-yielding stimuli randomly distributed in both spaces). As shown by plots and statistical analyses, the Towards Actor Group exploited mainly its near space, by selecting, at the end of the task, 70.51% of the stimuli in this space (compared to 67.41% at the beginning of the task), for a total of 69.37% of proximal stimuli selected all along the task. Instead, at the end of the task, the Towards Observer Group selected 60.90% of stimuli in the space near the observer (i.e., actor’s distal space, against 46.80% at the beginning of the task), for a total of 53.51% of distal stimuli selected all along the task. This effect can be explained by a statistical learning of reward-yielding stimuli location^49,50^ achieved through repeated motor interactions with the workspace. Statistical learning is associated with attentional mechanisms and it is known to alter stimulus priority within the spatial map of action space^51^ and to guide further search behaviour^52,53^. However, the effect of rewards distribution on the selection strategy emerged at different times for the two groups. It was present from the first block in the Towards Actor Group, while it occurred late in the task for the Towards Observer Group (from the 14th block on). These results can be explained by the presence of an effect of the social context, which has already been reported in the literature^41,42^. Actors tended to split the space in two and to act predominantly in their own proximal space in order not to invade the space of the observer. As further evidence for this interpretation, we can evoke the results found by Coello et al.^41^, who observed that participants started to exploit systematically the distal space quite early in the task (from the 3rd block on) when they had to perform the task alone.

Concerning actor’s PPS representation, results of the reachability-judgment task showed an extension of PPS representation when the distribution of reward-yielding stimuli was biased towards the actor’s distal space. No change was observed instead when the distribution of reward-yielding stimuli was biased towards the actor’s proximal space, nor when it was random across both spaces. These findings are in agreement with what reported in previous studies^41,42^ and corroborate the effect of statistical learning of rewards distribution on PPS representation. They also confirm that the effect of rewards on PPS representation is modulated by the presence of another person implied in the task^54,55^, which exert a combined and opposite effect. Indeed, it was shown that when rewards distribution was biased towards the actors’ proximal space, their PPS representation did not constrict, probably to allow the individual to maintain a safety buffer space in front of the confederate^42^.

As regards the observer’s performances, the main finding of the present study was the presence of dissociated effects of actors’ rewards on the observer’s PPS representation and exploitation. Concerning observers’ PPS representation, results showed an extension of PPS representation after observing actors getting more rewards in their proximal space (i.e., the distal space for the observer). No significant change in PPS representation was found after observing actors getting more rewards in their distal space (i.e., the proximal space of the observer). It is worth noting that these results are symmetrical with the pattern observed for the actor and can be explained in the same way, that is, by an implicit statistical learning of visual regularities, here consisting in rewards’ location, altering the spatial priority map^50,53,56^. In the case of the observers, statistical regularities are learnt from the outcomes of others’ actions instead of their own actions. Overall, these results echo previous findings showing that the PPS representation can be altered by the observation of conspecifics’ motor performances^45^. Surprisingly, we observed a constriction of PPS representation for observers in the Control group, for whom rewards were equally distributed in the distal and proximal spaces. This effect could be due to the fact that when rewards were equally distributed in space, actors explored the whole action space, a behaviour that could have been experienced by the observers as a violation of their own PPS. Alternatively, in such a situation, we could have expected an increase of observers’ PPS following the rewards obtained by the actor across the whole action space. Such a result was indeed observed by^41^, where both participants actively performed the task. Nevertheless, since we observed a reduction instead of an increase, we might conclude that when the individual is passively involved in a motor task, protecting oneself from social invasion becomes a prominent factor bypassing the effect of others’ action rewards. Finally, it is important to note that while in the Control group we observed a reduction of PPS for the observer (that we explained as a response to the violation of their own space), in the Towards Observer condition no significant change of PPS was observed. To explain this, we can speculate that in this condition, the effect of the invasion might have been counterbalanced or attenuated by the presence of more rewards in the space near the observers*.* Taken as a whole, the present findings suggest that others' rewards spatial distribution can be taken into account to adjust one’s own PPS representation, but that the implications of a co-action versus a cooperative social context must be more thoroughly studied in future researches in order to precisely disentangle their respective impact.

Finally, the other important result of the present study was the non-significant effect of actors’ rewards on the observers’ selection strategy and thus, PPS exploitation. A potential explanation for this non-significant effect could be that some participants switched their viewing perspective^57^. That is to say, if reward-yielding stimuli were mainly situated in the proximal space of the actor, participants could have thought that, symmetrically, rewards were located in the space near themselves. However, if this argument was correct, we should have observed, on the one hand, different within-group variability in Towards Actor and Towards Observer groups compared to the Control. On the other hand, we should have observed no effect on PPS representation. Yet, this was not the case. Therefore, this indicates that the explanation based on a switch of viewing perspective can be ruled out. A second explanation could be related to a potential decay of the statistical learning in the observers at the moment of acting. However, we found no significant change in the reachability thresholds when comparing the first and the second part of the reachability task in the posttest, suggesting that the effect of observing others’ action outcomes did not decay before the performance of the stimuli-selection task. Moreover, evidence from literature does not support this explanation either. Indeed, statistical learning seems a very stable and robust mechanism which proved to last at least 30 min^58^ and even up to 24 h^59^. As the delay between the observation and the performance of the stimuli-selection task was on average inferior to 15 min, the hypothesis of a decay of the implicit learning can thus be dismissed, although the temporal aspect of this decay would have to be studied in the future. Finally, the non-significant effect of others actions on the observers’ PPS exploitation could be due to the fact that, although implicitly learning rewards distribution from another’s actions affects the observer’s attentional and spatial maps of the visual workspace, motor experience is needed to fully embody this learning and its outcomes, and consistently orient the observer’s selection strategy. That is to say, we could speculate that observing others' action rewards would modify PPS representation (altering the visual priority map of space), but this effect would be insufficient to generalise to the organisation of motor experience in the PPS (altering the motor priority map of space). Although the present findings provide new insights into PPS processing, further studies would be needed to better understand the relationship between PPS representation and exploitation as well as the different factors that could influence it.

## Conclusions

The present study showed that observing another’s actions outcome in space has a dissociated effect on the observers’ PPS representation and exploitation. Action observation contributes to spatially localising reward-yielding stimuli in relation to the body, in order to build a suitable representation of one's own PPS. However, subsequent PPS exploitation, and therefore action selection, would rather require a personal motor experience of PPS. In conclusion, observing others' actions outcome would differently alter PPS representation (specifying the perceptual priority map of space) and PPS exploitation (specifying the motor priority map of space), a new framework that would require further empirical validations.

## Methods

### Participants

156 healthy and right-handed (mean laterality quotient = 0.85, SD = 0.18, Oldfield, 1971) participants (age range 18–35 years, M = 21.34, SD = 2.53; 100 females), took part in the experiment in exchange of course credits. They were recruited from the Psychology Department of the University of Lille (France), declared having no perceptual or motor troubles and had normal or corrected-to-normal visual acuity. Participants gave their consent after receiving an information letter about the experiment. The experimental protocol was conducted in accordance with the ethical principles of the Declaration of Helsinki^60^ and was approved by the University of Lille Institutional Ethics Committee (Ref. Number 2019-374-S77).

The required sample size was calculated a priori using the G*Power software (3.1.9.4). For this purpose, we chose the ANOVA-Repeated measures, within-between interaction module, which was the most suited one to our experimental design, considering the Session (Pretest, Posttest) as within-subjects factor and the Group (Control, Towards Actor, Towards Observer) as between-subjects factor. We chose a Cohen’s F expected effect size of 0.40 (large effect). This value was chosen on the basis of two elements. On the one hand, the fact that the effect size found by Coello et al.^41^ was *η*^2^*p*  = 0.20, corresponding to a Cohen’s *F* = 0.50 (large effect). On the other hand, the fact that weaker effects were expected because of the situation of observation used in the present paradigm. After choosing an expected effect size of 0.40, we ran a power analysis taking into consideration a power of 80% (and *α* = 0.05), which indicated that a sample of 23 participants per each condition would be required. In order to reach this goal, we decided to recruit 26 participants in each group, which allowed us to account for a possible attrition rate of 5% on all groups considered together (i.e., 3 participants to be excluded in a dataset).

### Apparatus, stimuli and procedure

**Figure 5:**
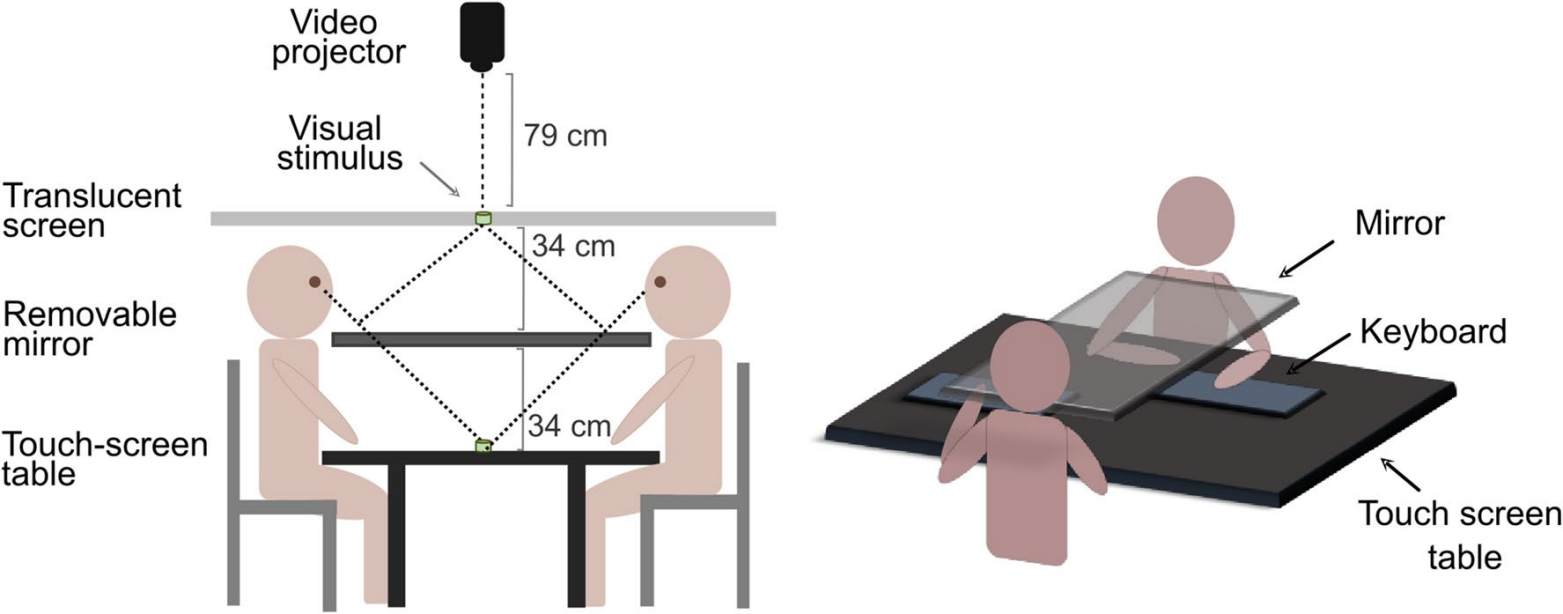
(**A**) Schematic representation of the experimental setting. During the reachability-judgment task, the video-projector projected an image on the mirror, through a translucent screen (which improved the sharpness of the image). This generated an optical projection effect, increasing the depth of the visual field and making the stimuli appear at the level of the touch-screen table. (**B**) Participants’ posture during the reachability-judgment task. The mirror hid participants’ hands and the keyboards used to provide the answers. During the task, the touch-screen table was covered by a black sheet, in order to avoid any interference from external luminous sources. Once the reachability-judgment task was completed, the mirror and the keyboards were displaced on the side, and the black sheet covering the touch-screen table removed.

Experimental setting, paradigm and procedure were based on^41,42^ studies. The experimental apparatus (see Fig. 5) consisted in a 40″ touch-screen table (Samsung SUR40, 109.5 × 70.74 cm), placed in the middle of a steel structure supporting a 30 cm × 100 cm horizontal rectangular mirror and a 200 × 150 cm horizontal translucent screen. In each experimental session, two participants sat face to face on each side of the touch-screen table. They performed a *reachability-judgment task* and a *stimuli-selection task.* Depending on the task, the stimuli were displayed by the touch-screen table (*stimuli-selection task*) or by a video-projector (*reachability-judgment task*) on the mirror, which displayed the stimuli as if they were located on the touch-screen table. The tasks were implemented using MATLAB software (R2017a).

#### Reachability-judgment task

In the *reachability-judgment task*, the stimuli consisted of 51 grey dots (1 cm diameter) on a black background projected on the mirror by the video-projector (Infocus 3926D) and through the translucent screen. Stimuli were presented one by one in a random order in the segment between participants’ foreheads, corresponding to a distance of 1 m (with an inter-stimuli gap of 2 cm and an inter-stimuli interval of 1.5 s). Accordingly, a target presented at 10 cm from the head of one participant corresponded to a target located at 90 cm from the head of the other participant. Each stimulus was displayed four times for a duration of 250 ms, providing a total of 204 trials (51 distances × 4 repetitions). During the task, the touch-screen table was covered by a black sheet in order to eliminate the effect of luminous sources on stimuli presentation. A short training session (5 trials) was performed at the beginning of the task and a short rest of 60 s was given halfway through the task. The two participants provided reachability estimates for each stimulus by pressing the left-arrow (reachable) and right-arrow (unreachable) keys of a keyboard with their left and middle fingers. Answer keys were counterbalanced across participants, meaning that after random assignment, half of the participants used the left-arrow to provide “reachable” responses (and the right-arrow to provide “unreachable” responses), while the other half used the right-arrow to provide “reachable” responses (and the left-arrow for “unreachable” ones).

#### Stimuli-selection task

In the *stimuli-selection task*, the stimuli consisted of 32 grey dots (2.7 cm diameter) displayed on a black background by the touch-screen table (1920 × 1080 px, active area: 88.56 × 49.81 cm). The stimuli were displayed following a non-visible distribution grid composed of 42 cells (6 rows × 7 columns, Fig. 1A). For each block of stimuli selections, 32 cells out of the 42 were randomly selected to contain the stimuli, which appeared at random positions according to the centre of the cell (varying from 0 to 60 pixels in the *x, y* directions). This allowed us to obtain a different pseudo-random stimuli configuration in each block. The stimuli were selected by touching them one by one with the right index finger. Once selected, stimuli changed colour from grey to either red or green (both colours had 50% of chances to occur). When the stimulus became green, a sound of clinking coins was played and participants obtained one point (reward-yielding stimulus). When it became red, a buzzing sound was played and participants obtained no point (no reward-yielding stimuli). Two digital counters located on the middle of the two proximal edges of the screen displayed the total number of points obtained and updated after each stimulus selection. Moreover, following the group assignment, the probability of finding a green, reward-yielding stimulus varied depending on the stimulus location on the touch-screen table (see Fig. 1B). In the Control group, it was 50% both in the space near the actor (rows 1, 2 and 3 of the grid) and in the space near the observer (rows 4, 5 and 6). In the Towards Actor group, it was 75% in the space near the actor and 25% in the space near the observer. In reverse, in the Towards Observer group, it was 75% in the space near the observer but 25% in the space near the actor. Finally, during the whole task, the actor and the observer had no right to communicate either verbally or non-verbally.

### Procedure

Participants were invited to sit around the table. Prior to the beginning of the experiment, the experimenter measured participants’ right arm length (i.e., the distance between the acromion bone and the tip of the middle finger) and adjusted the height of their chair so that their chin was 3 cm above the edge of the mirror. Following that, participants were randomly assigned either the role of the actor or the role of the observer. The task began with a first reachability-judgment task (pretest), performed by both the actors and the observers. During this task, the stimuli were presented on the mirror placed in between participants (see Fig. 5), which hid their arms from their view. The touch-screen table was covered by an opaque cloth to avoid that light sources could interfere with the execution of the task. Then, actors executed the stimuli-selection task, while observers were instructed to attentively observe them. During this phase, the mirror was moved to the side and the black cloth covering the table was removed, so as to allow participants to see the stimuli on the touch-screen table and their arms. Next, both the actors and the observers performed a second time the reachability-judgment task (the mirror and the black cloth were put back in place). Finally, the observer executed the stimuli-selection task, while the actor remained still.

During the reachability-judgment task, the actors and the observers received the same following instructions: *“A series of luminous dots will be successively presented on the table in front of you. For each dot displayed, you will have to estimate whether it seems reachable *(*or not*)* when imagining simply extending your right arm, without turning your shoulders or moving your body forward. You will use your left index and middle fingers to provide your answer on the keyboard. You will have to press—“Left” *(*or right, according to the counterbalancement rule reported earlier*)* when the luminous dot seems reachable and “Right” *(*or left) if it does not seem reachable. The dots will be presented during a very short delay. Therefore, try to answer as fast as possible, but try to make as few errors as possible. Your answer should be as instinctive as possible. Finally, please be careful not to move your head *(*forwards or backwards) during the whole task”.*

The stimuli-selection task was presented to the actors and the observers as a game that had to be played together. They received the following instructions: *“During this game, a set of stimuli will be randomly distributed on the touch-screen table in front of you. When selected, a stimulus will change its colour: if it turns green, you win 1 point, if it turns red, you win no point. When you select 12 stimuli, a new game round, that is, a new set of stimuli, will be displayed. During this game, each one of you will have a specific objective:”*

- Instructions given to the actor: “*As regards you, your task will be to select 12 consecutive stimuli during each game round. Your objective will be to try to find as many green stimuli as possible. To select a stimulus, you will have to click on it by using your right index finger. You will have to use only your right arm*.”
- Instructions given to the observer: “*In the meanwhile, your task will be to observe your partner’s performance. At the end, it will be your turn to play: you will have the possibility to make 17 supplementary game rounds, in order to obtain more points and increase the final score of the dyad. The aim of this game is to find as many green stimuli as possible, in order to obtain the highest score in collaboration with your partner! Try to beat the other dyads by achieving the highest score together! The dyad that will achieve the highest score will be rewarded with a surprise prize! Be careful, there is only one rule: during the whole game, you will not be allowed to communicate, neither verbally nor by gestures. Now, it is time to play!*”

### Data analysis

#### Reachability-judgment task

As concerns the reachability-judgment task*,* we computed participants’ reachability thresholds (used as a proxy of PPS representation) by applying logistic regression. We used a maximum likelihood fit procedure based on second-order derivatives (quasi-Newton method) to find the logit model that best fitted the distribution of dichotomous responses (reachable/unreachable) provided by the participant to each of the 51 stimuli distances^39^. The logit model was obtained through Eq. (1):

1 $$Y=\frac{{exp}^{\left(\alpha +\beta X\right)}}{1+{exp}^{\left(\alpha +\beta X\right)}}$$

In the above equation, Y is reachable/unreachable answer provided by the participant, X the distance at which each one of the stimuli was presented, and − $\frac{\alpha }{\beta }$ the inflection point of the curve, denoting the critical value of X at which the transition from “reachable” to “unreachable” responses occurred (i.e., the probability of responding “unreachable” changed from below to above 0.5). Therefore, − $\frac{\alpha }{\beta }$ corresponds to the reachability threshold. Individual reachability thresholds were corrected by subtracting participant’s arm length to the − $\frac{\alpha }{\beta }$ value. Reachability thresholds were computed separately for the pretest and the posttest sessions.

#### Stimuli-selection task

As concerns the stimuli-selection task, we assessed participants’ exploration strategy (PPS exploitation) by computing:

1. The mean number of stimuli selected *across all blocks* in the space near the observer (when analysing the actor’s performance) or in the space near the actor (when analysing the observer’s performance)*,* in order to assess groups’ tendency to explore the space associated with higher number of reward-yielding stimuli.
2. The mean number of stimuli selected in the *first three and last three blocks* only, in the spaces near the observer or near the actor, in order to test whether the groups changed their selection strategy during the task.
3. The mean number of stimuli selected in *each block*, in the spaces near the observer or near the actor, to identify the precise moment at which the change in selection strategy occurred.
4. The mean number of reward-yielding stimuli obtained in the spaces near the observer or near the actor, to check the validity of the experimental design.

### Statistical analysis

Statistical analyses were carried out on R version 3.6.1 (R Core Team, 2019) and R Studio version 1.1.456. Prior to the main statistical analysis, we checked for the presence of outliers using the median absolute deviation (MAD) method. In order to remove extreme datapoints and to concurrently keep the amount of discarded data below 5%, the threshold for outliers’ rejection was set as the difference from the median ± 2.75 times the MAD^61^. This threshold corresponds to a very to moderately conservative criterion that, despite leaving the presence of mild outliers, complies with the trend in psychology studies to keep as much inter-individual variability as possible. Outliers’ analysis was conducted only in the reachability-judgment task, as it is more sensitive to extreme values, and on the posttest–pretest difference in reachability threshold, as it is at the core of our research hypothesis. Outliers’ analysis was carried out separately for the actor and the observer datasets, and separately for each group. We removed 2 outliers in the actor dataset and 2 in the observer one. Two additional participants (1 in each dataset) were also excluded, as they did not correctly execute the task, providing no exploitable responses.

All parametric one-way and two-way ANOVAs were carried out using the function anova_test. Following ANOVA, parametric simple effect tests as well as post-hoc multiple comparisons were performed using the function pairwise_t_test. In case of violation of the homoscedasticity assumption, we carried out a one-way Welch’s ANOVA for independent groups using the function welch_anova_test. In this case, simple effects were tested through the Wilcoxon signed-rank test, performed with the functions wilcox_test and post-hoc multiple comparisons through the Games-Howell test, using the function games_howell_test. In case of violation of the homoscedasticity and normality assumptions, we performed a Kruskal–Wallis test, a non-parametric equivalent of one-way ANOVA for independent groups designs, by using the functions Kruskal_test. Post-hoc multiple comparisons following Kruskal–Wallis test were performed by the means of the Dunn’s test, using the function dunn_test. All the functions aforementioned were part of the package rstatix version 0.7.0^62^. As concerns effect sizes, we computed partial eta-squared (*η*^2^*p*, using the function anova_test), except for the non-parametric tests for which effect sizes were calculated using the indexes specifically conceived for these tests (*r* for the Wilcoxon signed-rank test using the function wilcox_effsize, *ε*^2^_*H*_ for Kruskal–Wallis using the function kruskal_effsize and *ω*^2^ for one-way Welch’s ANOVA).

In the stimuli-selection task, the performances of the Towards Actor and the Towards Observer Groups were compared to the Control Group for each block of trials by means of permutation-based multiple comparisons (based on 9999 Monte-Carlo resampling). Permutation-based multiple comparisons were run using the independence_test function of the package coin version 1.4-2 (see^63,64^ for a description of the permutation method employed). Being a resampling technique, permutation-based multiple comparisons allow the control for Type-I error and for the occurrence of false positives^65^. Therefore, no correction of p values was applied as it was not needed.

For parametric tests, the presence of a violation of the normality assumption was assessed by using the Shapiro–Wilk test and checking Q–Q plots (to check for a major violation of the normality assumption that could compromise the validity of ANOVA). The assumption of variances homogeneity for between-subjects factors was verified by means of the Levene’s test, while the assumption of variances homogeneity for the differences between within-subjects groups (i.e., sphericity assumption) was verified by the Mauchly’s test (Greenhouse–Geisser sphericity correction was applied in case of violation). The assumption of covariances homogeneity was verified by using the Box’s M test. Significance threshold was set at α = 0.05, except for the tests verifying the assumptions of homoscedasticity and homogeneity of the variance–covariance matrix (for which α = 0.10), for the Box’s M test (for which *α* = 0.001) and for the post-hoc multiple comparisons, for which p values were adjusted using Benjamini–Hochberg procedure.

As concerns the application of the Benjamini–Hochberg correction procedure, in the present study we realised 10 tests to adress 10 different questions (4 for Actors, namely: the change between pretest and posttest in reachability thresholds for each group, the difference between the three groups in the stimuli-selection task across all blocks, the comparison within each group in the stimuli selection between the first three and the last three blocks, the regression on the number of stimuli selected across all blocks; and 6 for Observers, namely: the same as for the actor, plus the test on the decay effect of observing actor's performance and the comparison of the first three blocks only to see whether the effect of observing actor's performance is present at least at the beginning). For each of these 10 tests, there are 3 p values, 1 per each comparison in the secondary tests, which need to be corrected. Therefore, this corresponds to a total of 10 × 3 = 30 secondary tests p values. The Benjamini–Hochberg correction procedure was applied considering these 30 secondary tests/p values (see the Table 1 in Supplementary Material). To note, although all the 30 secondary tests were considered for the correction (in order to make the correction independent from the number of significant results) only the secondary tests following significant ANOVAs were interpreted in the text.

## Supplementary Information

Supplementary Table 1.

## Data Availability

The datasets generated and analysed in the current study are available from the corresponding author (Yann Coello) upon request.

## Code availability

The R and MATLAB scripts used for statistical analysis and figures are available from the authors upon request.
