# Supplementary material for: Paying attention to the outcome of others' actions has dissociated effects on observer's peripersonal space representation and exploitation
Source: Sci Rep. 2023 Jun 22;13:10178. doi: 10.1038/s41598-023-37189-8 (PMC10287734; doi:10.1038/s41598-023-37189-8)
Supplement: Supplementary file 1 — Supplementary Table 1. [file 41598_2023_37189_MOESM1_ESM.docx]

**Supplementary Material**

|  | Dataset | Task | Variable | Test | Comparison | Raw *p* | Adjusted *p* |
| --- | --- | --- | --- | --- | --- | --- | --- |
| 1 | Actor | Reachability-judgment | Reachability threshold | Simple effect of Session (pairwise_t_test) | Posttest vs pretest (CT) | 0.402 | 0.5628 |
| 2 | Actor | Reachability-judgment | Reachability threshold | Simple effect of Session (pairwise_t_test) | Posttest vs pretest (TA) | 0.818 | 0.904 |
| 3 | Actor | Reachability-judgment | Reachability threshold | Simple effect of Session (pairwise_t_test) | Posttest vs pretest (TO) | 0.012 * | 0.045 * |
| 4 | Actor | Stimuli-selection | Mean number of distal stimuli (All blocks) | Dunn Post Hoc | CT vs TA | 0.001** | 0.01 * |
| 5 | Actor | Stimuli-selection | Mean number of distal stimuli (All blocks) | Dunn Post Hoc | CT vs TO | 0.092 | 0.176 |
| 6 | Actor | Stimuli-selection | Mean number of distal stimuli (All blocks) | Dunn Post Hoc | TA vs TO | 0.000000935 *** | 2.805e-05 *** |
| 7 | Actor | Stimuli-selection | Mean number of distal stimuli (First 3-Last 3 blocks) | Simple Effect of Block (Wilcox_ttest) | First 3 vs Last 3 blocks (CT) | 0.647 | 0.799 |
| 8 | Actor | Stimuli-selection | Mean number of distal stimuli (First 3-Last 3 blocks) | Simple Effect of Block (Wilcox_ttest) | First 3 vs Last 3 blocks (TA) | 0.603 | 0.791 |
| 9 | Actor | Stimuli-selection | Mean number of distal stimuli (First 3-Last 3 blocks) | Simple Effect of Block (Wilcox_ttest) | First 3 vs Last 3 blocks (TO) | 0.009 ** | 0.045 * |
| 10 | Actor | Stimuli-selection | Mean number of distal stimuli (per block) | Regression | CT | 0.071 | 0.166 |
| 11 | Actor | Stimuli-selection | Mean number of distal stimuli (per block) | Regression | TA | 0.234 | 0.378 |
| 12 | Actor | Stimuli-selection | Mean number of distal stimuli (per block) | Regression | TO | 0.000674 *** | 0.01 * |
|  |  |  |  |  |  |  |  |
| 13 | Observer | Reachability-judgment | Reachability threshold | Simple effect of Session (pairwise_t_test) | Posttest vs pretest (CT) | 0.003 ** | 0.022 * |
| 14 | Observer | Reachability-judgment | Reachability threshold | Simple effect of Session (pairwise_t_test) | Posttest vs pretest (TA) | 0.011 * | 0.045 * |
| 15 | Observer | Reachability-judgment | Reachability threshold | Simple effect of Session (pairwise_t_test) | Posttest vs pretest (TO) | 0.81 | 0.904 |
| 16 | Observer | Reachability-judgment | Reachability threshold (First half of stimuli vs Second half of stimuli) | Simple effect of Split (wilcox_test) | First half vs Second half (CT) | 0.252 | 0.378 |
| 17 | Observer | Reachability-judgment | Reachability threshold (First_Second Half) | Simple effect of Split (wilcox_test) | First half vs Second half (TA) | 0.007 ** | 0.042 * |
| 18 | Observer | Reachability-judgment | Reachability threshold (First_Second Half) | Simple effect of Split (wilcox_test) | First half vs Second half (TO) | 0.09 | 0.176 |
| 19 | Observer | Stimuli-selection | Mean number of distal stimuli (All blocks) | Student’ t test for unpaired samples | CT vs TA | *Not shown as main test not significant* | *Not shown as main test not significant* |
| 20 | Observer | Stimuli-selection | Mean number of distal stimuli (All blocks) | Student’ t test for unpaired samples | CT vs TO | *Not shown as main test not significant* | *Not shown as main test not significant* |
| 21 | Observer | Stimuli-selection | Mean number of distal stimuli (All blocks) | Student’ t test for unpaired samples | TA vs TO | *Not shown as main test not significant* | *Not shown as main test not significant* |
| 22 | Observer | Stimuli-selection | Mean number of distal stimuli (First 3 blocks) | Student’ t test for unpaired samples | CT vs TA | *Not shown as main test not significant* | *Not shown as main test not significant* |
| 23 | Observer | Stimuli-selection | Mean number of distal stimuli (First 3 blocks) | Student’ t test for unpaired samples | CT vs TO | *Not shown as main test not significant* | *Not shown as main test not significant* |
| 24 | Observer | Stimuli-selection | Mean number of distal stimuli (First 3 blocks) | Student’ t test for unpaired samples | TA vs TO | *Not shown as main test not significant* | *Not shown as main test not significant* |
| 25 | Observer | Stimuli-selection | Mean number of distal stimuli (First 3-Last 3 blocks) | Simple effect of Block (pairwise_t_test) | First 3 vs Last 3 blocks (CT) | *Not shown as main test not significant* | *Not shown as main test not significant* |
| 26 | Observer | Stimuli-selection | Mean number of distal stimuli (First 3-Last 3 blocks) | Simple effect of Block (pairwise_t_test) | First 3 vs Last 3 blocks (TA) | *Not shown as main test not significant* | *Not shown as main test not significant* |
| 27 | Observer | Stimuli-selection | Mean number of distal stimuli (First 3-Last 3 blocks) | Simple effect of Block (pairwise_t_test) | First 3 vs Last 3 blocks (TO) | *Not shown as main test not significant* | *Not shown as main test not significant* |
| 28 | Observer | Stimuli-selection | Mean number of distal stimuli (per block) | Regression | CT | 0.118 | 0.206 |
| 29 | Observer | Stimuli-selection | Mean number of distal stimuli (per block) | Regression | TA | 0.871 | 0.914 |
| 30 | Observer | Stimuli-selection | Mean number of distal stimuli (per block) | Regression | TO | 0.921 | 0.921 |

**Table 1. Tests performed, raw and adjusted p-values.** The adjusted *p*-values (p.adj column) were obtained after applying Benjamini-Hochberg correction on 30 secondary tests *p*-values. 9 secondary tests *p*-values were included in the correction, but not reported nor interpreted in the main text, as the primary main tests were not significant (see also Method section). CT: Control group; TA: Towards Actor group; TO: Towards Observer group**.** **p* < .050, ***p* < .010, ****p* < .001.
